# Ethnopharmacological exploration and isolation of HIV-1 latency-reversing agents from Sudanese medicinal plants

**DOI:** 10.3389/fphar.2025.1587128

**Published:** 2025-08-06

**Authors:** Khaled M. Elamin, Naoki Kishimoto, Teppei Kawahara, Sara Mustafa Idris Elbashir, Tae Yasutake, Mikiyo Wada, Yuki Hitora, Maha Kordofani, Wadah Osman, Mustafa Idris Elbashir, Shogo Misumi

**Affiliations:** ^1^ Global Center for Natural Products Research, Faculty of Life Sciences, Kumamoto University, Kumamoto, Japan; ^2^ Department of Environmental and Molecular Health Sciences, Faculty of Medical and Pharmaceutical Sciences, Kumamoto University, Kumamoto, Japan; ^3^ Department of Natural Medicines, Graduate School of Pharmaceutical Sciences, Kumamoto University, Kumamoto, Japan; ^4^ Department of Botany, Faculty of Science, University of Khartoum, Khartoum, Sudan; ^5^ Department of Pharmacognosy, Faculty of Pharmacy, Prince Sattam Bin Abdulaziz University, Al-kharj, Saudi Arabia; ^6^ Department of Pharmacognosy, Faculty of Pharmacy, University of Khartoum, Khartoum, Sudan; ^7^ Department of Biochemistry, Faculty of Medicine, University of Khartoum, Khartoum, Sudan

**Keywords:** Sudanese medicinal plants, HIV-1/AIDS, latency-reversing agents, anti-HIV drugs, Sudanese traditional healers

## Abstract

HIV-1 infection remains a major health challenge, especially in resource-limited settings such as Sudan, where traditional medicine is widely practiced for managing infectious diseases, including HIV/AIDS. In this study, we selected ten Sudanese medicinal plants traditionally used to treat immune-related and infectious diseases. The selection was based on ethnobotanical reports and local knowledge of HIV/AIDS-related treatments. Crude extracts were prepared using either absolute methanol or 50% ethanol via maceration, resulting in a total of 20 extracts. The extracts were then screened for HIV-1 latency reversal using a luciferase reporter assay in TZM-bl cells. The 50% ethanolic extract of *G. kraussiana* showed the highest LTR activation (EC_50_ = 3.75 μg/mL) with no significant cytotoxicity observed. Bioactivity-guided fractionation of the *Gnidia kraussiana* extract led to the isolation of gnidilatidin, a daphnane-type diterpenoid, using ultrahigh-performance liquid chromatography coupled with high-resolution mass spectrometry (UHPLC-HRMS). Gnidilatidin demonstrated potent latency-reversing activity (EC_50_ = 5.49 nM in J-Lat 10.6 cells) and downregulated CD4 and CXCR4, suggesting enhanced inhibition of HIV-1 entry. This study supports the ethnopharmacological relevance of *G. kraussiana* and validates its traditional use. It also identifies gnidilatidin as a promising lead compound for HIV-1 latency-reversal-based strategies. Further studies are needed to optimize its pharmacological profile and further elucidate its therapeutic potential, particularly as part of an optimized combination regimen with combination antiretroviral therapy (cART).

## 1 Introduction

Since the advent of combination antiretroviral therapy (cART), there has been a significant decrease in HIV-1-related morbidity and mortality among treated patients (Tseng et al., 2015). However, cART is insufficient to achieve a cure because it reduces the blood viral load but does not eliminate latent HIV-1-infected cells. Interruption of cART can result in an increase in viral load in the peripheral blood, highlighting the insufficiency of cART to achieve a cure. This limitation has led to an intensive worldwide effort to develop new therapeutic strategies to eradicate latently HIV-1-infected cells. One proposed strategy to achieve a cure is called “shock and kill,” which aims to reactivate latent HIV-1 to be recognized and destroyed by immune cells (Darcis et al., 2017). Consequently, significant research efforts are currently focused on finding new latency-reversing agents (LRAs) capable of reversing HIV-1 latency (Ait-Ammar et al., 2020). Although several LRAs have been investigated in clinical trials, there is no LRA that has consistently reduced the amount of viral reservoir carried in humans (Boucau et al., 2020; Fidler et al., 2020). Therefore, it is essential to continue the search for candidate LRAs from medicinal plants and identify components with good “shock and kill” properties, especially in areas with limited therapeutic resources.

Several natural compounds have been reported to have potential as LRAs. Prostratin, a phytoconstituent derived from the bark of *Homalanthus nutans*, is an excellent example of a natural product that produces a potent LRA (Johnson et al., 2008). In addition, we previously reported that some natural compounds are promising LRAs, such as gnidimacrin, stelleralide A, and wikstroelide A (El-Desoky et al., 2022). The findings indicate that natural resources represent a significant target for the exploration of new LRA candidates. Traditional medicine practices with natural resources are very popular in many African countries. Natural products from medicinal plants are used to treat many diseases including HIV/AIDS (Okello and Musisi, 2015). Despite massive global efforts, the HIV/AIDS disease burden remains disproportionately higher in low- and middle-income countries (LMICs) than in high-income countries (Shao and Williamson, 2012). One of the major barriers to receiving effective cART in LIMICs is the high cost of these treatment regimens, which results in their limited accessibility for HIV-1-positive patients living in LMICs (Haakenstad et al., 2019). Currently, about 34% of HIV-1-positive individuals in Eastern and Southern Africa and 60% of HIV-1-positive individuals in Western and Central Africa are not receiving any treatment (UNAIDS, 2017; Jewell et al., 2020). Therefore, focusing on traditional medicine and finding LRAs and anti-HIV drugs in natural products available in local environments can help avert a public health crisis.

The evolution of traditional medicine in Sudan has been deeply influenced by the rich cultural heritage of diverse ethnic groups from Africa and the Middle East over the course of many centuries (El Safi, 2007). A comprehensive analysis of Sudan’s floral diversity has identified 3,137 species of flowering plants, classified into 170 families. Notably, over 136 of these species are used as medicinal plants highlighting the profound cultural and therapeutic significance of botany in Sudan (Khalid et al., 2012). Current data demonstrate that traditional medicine, particularly the use of medicinal plants, has historically served as a primary treatment modality for a wide range of conditions, including diabetes, hypertension, infectious diseases, autoimmune disorders, respiratory ailments, and gastrointestinal issues (Elbashir et al., 2018). Despite the identification of numerous bioactive compounds, such as flavonoids, saponins, alkaloids, steroids, terpenes, tannins, fatty acids, and essential oils in these plants, there is a lack of conclusive clinical studies to formally establish their efficacy as therapeutic agents in Sudan. Given the centuries-old tradition of using indigenous plant-based remedies for therapeutic purposes, it became essential to systematically evaluate the efficacy of certain traditional medicinal plant preparations in treating HIV/AIDS.

Sudan has a longstanding tradition of herbal medicine, with many traditional healers claiming success in treating infectious and immune-related diseases, including HIV/AIDS. Between 2016 and 2020, we conducted several workshops and scientific meetings with experienced herbalists in Khartoum to explore commonly used medicinal plants. Through these collaborative efforts, we identified a group of plants frequently employed in managing immune-related conditions. Illustrating from ethnobotanical knowledges and surveys, we selected ten representative species (Table 1) for further scientific evaluation of their potential as latency-reversing agents (LRAs) against HIV-1.

**TABLE 1:** List of Sudanese medicinal plants used by traditional healers in Sudan for the treatment of certain diseases which we evaluated for their Latency Reversing Activity (LRA).

Scientific name	Family	Voucher number	Extracted part	Traditional uses
*Anticharis senegalensis* (Walp.) Bhandari	Scrophulariaceae	1–3/8/2020	Roots	Prescribed for the treatment of Autoimmune diseases, and HIV/AIDS. It is claimed to be effective for treatment of asthma and COVID-19.
*Catunaregam nilotica* (Stapf) Tirveng.	Rubiaceae	1–4/8/2020	Stems	Prescribed for treatment of autoimmune diseases such as Lupus. It is also claimed to be effective for treatment of asthma and COVID-19.
*Fagonia cretica* L. *(*Zygophyllum creticum* (L.) Christenh. & Byng)	Zygophyllaceae	1–7/8/2020	Shrub	Prescribed to boost immunity in immunocompromised patients and for the treatment of HIV-1.
*Gnidia chrysantha* (Solms ex Schweinf.) Gilg	Thymelaeaceae	1–8/8/2020	Roots	Prescribed for treatment of asthma, and to relief respiratory system allergy.
*Gnidia kraussiana* Meisn *(*Lasiosiphon kraussianus* (Meisn.) Meisn.)	Thymelaeaceae	1–9/8/2020	Roots	Prescribed for treatment of HIV-1 Patients, syphilis and as inhalation to relief COVID-19 symptoms.
*Grewia villosa* Willd.	Malvaceae	1–2/8/2020	Stems	Prescribed for the treatment of sexually transmitted diseases, Hepatitis B and Hepatitis C virus
*Jatropha curcas* L.	Euphorbiaceae	1–5/8/2020	Seeds	Prescribed for the treatment of HIV/AIDS patients, sexual disability, and GIT symptoms.
*Leptadenia arborea* (Forssk.) Schweinf.	Apocynaceae	1–3/8/2020	Twigs	Prescribed to boost the immunity of immunocompromised patients and Hepatitis B virus infected people.
*Leptadenia pyrotechnica* (Forssk.) Decne.	Apocynaceae	1–6/8/2020	Aerial Part	Prescribed for the treatment of viral infection diseases such as Hepatitis B and Hepatitis C
*Maerua pseudopetalosa* (Gilg & Gilg-Ben.) DeWolf	Capparaceae	1–1/8/2020	Roots	Prescribed for treatment Diabetes, hypertension, and sexually transmitted diseases.

*Following recent updates to the names of two species, we present the new names as well.

In this study, we critically assessed the therapeutic potential of these plants in the context of HIV-related diseases at the laboratory level. Considering the extensive use of traditional medicine in Sudan, this study aimed to scientifically investigate the anti-HIV-1 efficacy of medicinal plants used in Sudanese traditional remedies. Utilizing ethnobotanical knowledge, we identified ten plants commonly employed in the management of immune-related and infectious diseases. As a result, 20 crude extracts derived from these plants were subjected to *in vitro* assays to evaluate their efficacy in reversing HIV-1 latency.

## 2 Materials and methods

### 2.1 Ethnobotanical study and collection of medicinal plants

The ethnobotanical study and collection of medicinal plants were conducted in accordance with the principles of the Convention on Biological Diversity (CBD) and the Nagoya Protocol on Access and Benefit Sharing (ABS). Prior informed consent (PIC) was obtained orally from Sudanese traditional healers during community-engaged workshops conducted in Khartoum. These consultations were facilitated and recorded under the supervision of the University of Khartoum and the Ministry of Higher Education, Sudan. Voucher specimens for all plant species were deposited at the Herbarium of the Department of Botany, University of Khartoum (see Table 1). No endangered species listed under CITES or the IUCN Red List were collected during the study. The present study adhered to the Four Pillars of Best Practice in Ethnopharmacology, which mandate ethical compliance and respect for the knowledge systems of indigenous and local communities. The initiative encompasses collaborative authorship with Sudanese researchers, capacity-building at the local level, and the recognition of herbalists within the community who contributed to the selection of plants. It is hereby confirmed that no material was exported without appropriate documentation, and that the research was conducted under a valid Memorandum of Understanding (MoU) between Kumamoto University and the University of Khartoum, thereby fostering equitable scientific benefit.

### 2.2 Preparation of medicinal plant extracts

Ten medicinal plant species were selected based on their frequent use in traditional Sudanese medicine for immune-related and infectious diseases, as listed in Table 1. All plant species were identified by an expert taxonomist (Professor Maha Kordofani) from the Herbarium of the Department of Botany, Faculty of Sciences, University of Khartoum. The plants were dried at 40°C using an electric oven. Each plant part (10 g) as mentioned in Table 1 was extracted using two solvents, absolute methanol and 50% ethanol, chosen to maximize the extraction of a broad range of polar and semi-polar phytochemicals. Maceration was performed by soaking the powdered material in 100 mL of solvent with continuous shaking at room temperature for 24 h. Filtrates were evaporated under reduced pressure using a rotary evaporator set at 45°C. This process ensured complete removal of solvents prior to DMSO dissolution. In total, 20 extracts were obtained. Finally, each extract was dissolved in DMSO at a concentration of 20 mg/mL and stored at −20°C for the evaluation of the cytotoxicity and transcriptional activation from HIV-1 LTR.

### 2.3 Cell culture

TZM-bl cells, which express luciferase and beta-galactosidase genes under the control of the HIV-1 LTR promoter, were maintained at 37°C in DMEM supplemented with 10% fetal bovine serum (FBS) containing 100 IU/mL penicillin and 100 μg/mL streptomycin, and 50 μg/mL gentamicin. J-Lat 10.6 cells, which are a widely accepted model for HIV-1 latent infection and express GFP by reactivation, were maintained at 37°C in RPMI supplemented with 10% FBS containing 100 IU/mL penicillin and 100 μg/mL streptomycin, and 50 μg/mL gentamicin (Jordan et al., 2003). These cells were obtained through the NIH AIDS Reagent Program. Jurkat cells were maintained at 37°C in RPMI supplemented with 10% FBS containing 100 IU/mL penicillin and 100 μg/mL streptomycin, and 50 μg/mL gentamicin.

### 2.4 Evaluation of transcriptional activation from HIV-1 LTR

Cytotoxicity of the 20 plant extracts was assessed in TZM-bl cells, and that of gnidilatidin was assessed in J-Lat 10.6 cells, using the Cell Counting Kit-8 (Dojindo Laboratories, Japan) and cell viability of J-Lat 10.6 cells was determined using a LIVE/DEAD^®^ Cell Viability Kit (Thermo Fisher Scientific K.K.), respectively. The latency-reversing activity of 20 extracts derived from ten Sudanese medicinal plants was evaluated in TZM-bl cells. The cells were treated for 24 h with each extract at concentrations of 1 and 100 μg/mL. Additionally, fractions obtained from *G. kraussiana* extract were evaluated in TZM-bl cells at dilution factors of 1:200, 1:2000, and 1:20000. The latency-reversing activity of gnidilatidin was evaluated in J-Lat 10.6 cells. Cells were treated for 24 h with gnidilatidin over a concentration range of 0.1–1,000 nM, and the percentage of GFP-positive cells was measured by flow cytometry. EC_50_ values for latency-reversing activity were determined and plotted using GraphPad Prism software (version 9).

### 2.5 Chemical fractionation

All fractions were obtained by a solid phase extraction (SPE) technique. Briefly, *G. kraussiana* extract was adjusted to a final concentration of 1 mg/mL at a total volume of 1 mL. Then, the extract sample was loaded in Isolute^®^ ENV+ (Biotage, Sweden). After loading the extract sample on the column, fractions were eluted gradually with water, 50% MeOH, and finally, 100% MeOH to obtain a total of four fractions. All fractions were dried overnight and 20 µL of DMSO was added to each fraction. Finally, the fractions were subjected to chemical identification and biological assessments.

### 2.6 UHPLC-HRMS analysis

Ultrahigh-performance liquid chromatography–high-resolution mass spectrometry (UHPLC-TOFMS) metabolite profiling was conducted on the extract derived from *G. kraussiana,* its fractions*,* and the standard compound gnidilatidin. Analyses were performed using a Xevo G2-XS QTof mass spectrometer (Waters, Milford, MA, United States) equipped with an electrospray ionization (ESI) source. The ESI conditions were as follows: capillary voltage, 3 kV; cone voltage, 40 V; source temperature, 150°C; desolvation temperature, 600°C; cone gas flow, 50 L/h; and desolvation gas flow, 1,200 L/h. Detection was performed in the positive ion mode in an m/z range of 100–2,000 Da with a scan time of 0.2 s. The separation was performed on an Acquity BEH C18 UPLC column (100 mm × 2.1 mm i.d.; 1.7 μm, Waters) using an optimized gradient (MeCN and H_2_O both containing 0.1% formic acid) of 5%–100% MeCN in 5 min. The flow rate was set to 0.8 mL/min, the temperature to 55°C, and the injection volume to 2 μL. Gnidilatidin used as a standard substance was derived from MCE^®,^ Inc. and had a purity of 99.47%.

### 2.7 Flow cytometry

The percentage of stimulated GFP-positive J-Lat 10.6 cells or the surface expression levels of HIV-1 entry receptors were determined by CytoFLEX (Beckman Coulter). To determine GFP-positive cells in J-Lat 10.6 cells, gnidilatidin-treated cells were stained with LIVE/DEAD™ Fixable Far Red Dead Cell Stain Kit (Thermo Fisher Scientific) and then fixed with 2% paraformaldehyde. To determine HIV-1 entry receptors in TZM-bl cells and Jurkat cells, compound-treated cells were stained with propidium iodide (Merck KGaA), a PE/cyanine7 anti-human CD4 antibody (BioLegend^®^), an Alexa Fluor^®^ 488 anti-mouse CD195 (CCR5) antibody (BioLegend^®^) and an APC anti-human CD184 (CXCR4) antibody (BioLegend^®^), respectively. The collected data were analyzed using Kaluza (BECKMAN COULTER).

## 3 Results

### 3.1 Sudanese medicinal plants contain LRA candidates

**FIGURE 1:**
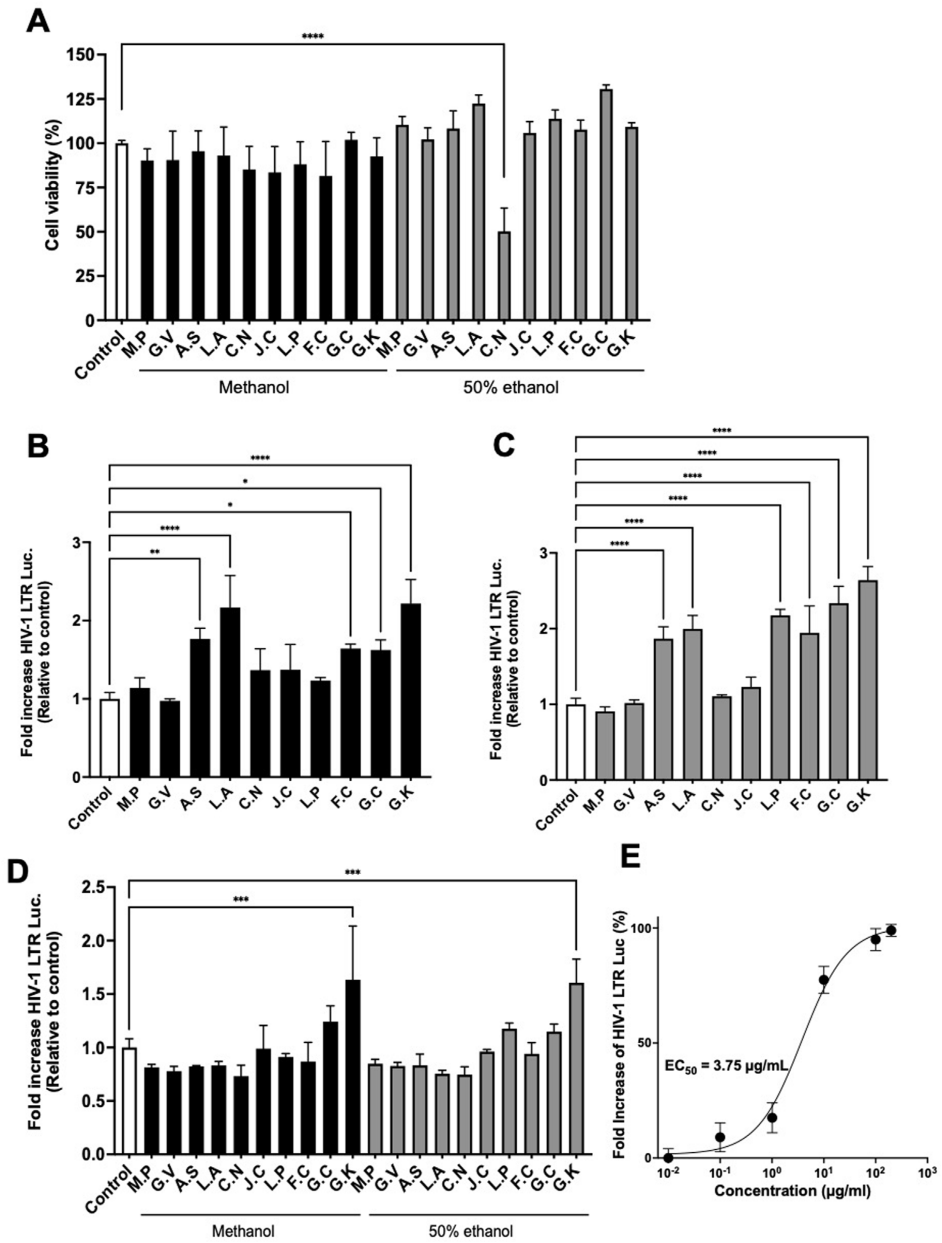
Extracts of Sudanese medicinal plants induce HIV-1 LTR transcriptional activity. **(A)** Cytotoxicity of extracts. TZM-bl cells were treated with 100 μg/mL of each extract. **(B)** Transcriptional activity from HIV-1 LTR induced by methanolic extracts. TZM-bl cells were treated with 100 μg/mL of each extract. **(C)** Transcriptional activity from HIV-1 LTR induced by 50% ethanolic extracts. TZM-bl cells were treated with 100 μg/mL of each extract. **(D)** Transcriptional activity from HIV-1 LTR induced by both methanolic and 50% ethanolic extracts at 1 μg/mL. **(E)** Dose-response curve and calculated EC_50_ value for the 50% ethanolic extract of *Gnidia kraussiana*. For all panels, each extract was dissolved in DMSO and prepared to a final concentration of 0.5% DMSO in the culture medium. Control corresponds to 0.5% DMSO. M.P, *Maerua pseudopetalosa*; G.V, *Grewia villosa*; A.S, *Anticharis senegalensis*; L.A, *Leptadenia arborea*; C.N, *Catunaregam nilotica*; J.C, *Jatropha curcas* L.; L.P, *Leptadenia pyrotechnica*; F.C, *Fagonia cretica* L.; G.C, *Gnidia chrysanth*a; G.K, *Gnidia kraussiana*. Statistical analysis was performed using one-way ANOVA with Dunnett’s multiple comparisons test (GraphPad Prism). *P < 0.05, **P < 0.01, ***P < 0.001, ****P < 0.0001. Each value represents the mean ± S.D of three experiments.

Based on the information from Sudanese traditional healers, to confirm whether Sudanese medicinal plants contain active substances that could act as LRAs, 20 prepared extracts from the plant materials shown in Table 1 were evaluated for their activity in inducing transcription from the HIV-1 LTR. As shown in Figure 1A, most extracts exhibited no significant cytotoxicity at 100 μg/mL, with the exception of the 50% ethanol extract of *Catunaregam nilotica*, which showed approximately 50% cytotoxicity at this concentration. To identify the most effective extract, TZM-bl cells were treated with 100 μg/mL of methanolic and 50% ethanolic extracts, which were prepared and tested separately from each plant. The methanolic extracts from *Anticharis senegalensis, Leptadenia arborea, Fagonia cretica, Gnidia chrysantha, and Gnidia kraussiana* exhibited significant transcriptional activity for HIV-1 LTR at a dose of 100 μg/mL compared with the control (Figure 1B). In addition, 50% ethanolic extracts from *A. senegalensis, L. arborea, F. cretica, Leptadenia pyrotechnica, G. chrysantha, and G. kraussiana* also exhibited significant transcriptional activity from HIV-1 LTR at a dose of 100 µg/mL compared with the control (Figure 1C). For further selection, TZM-bl cells were treated with each extract at a concentration of 1 μg/mL, and *G. kraussiana* extract was shown to be the most effective (Figure 1D). Therefore, we determined that a 50% ethanolic extract of *G. kraussiana* was the most effective candidate. The EC_50_ of the 50% ethanolic extract of *G. kraussiana* in this assay system was 3.34 μg/mL (Figure 1E). This finding represents the initial report of the potential of *G. kraussiana* extracts from Sudan to stimulate high transcriptional activity from HIV-1 LTR.

### 3.2 The active compound in *Gnidia kraussiana* is gnidilatidin

**FIGURE 2:**
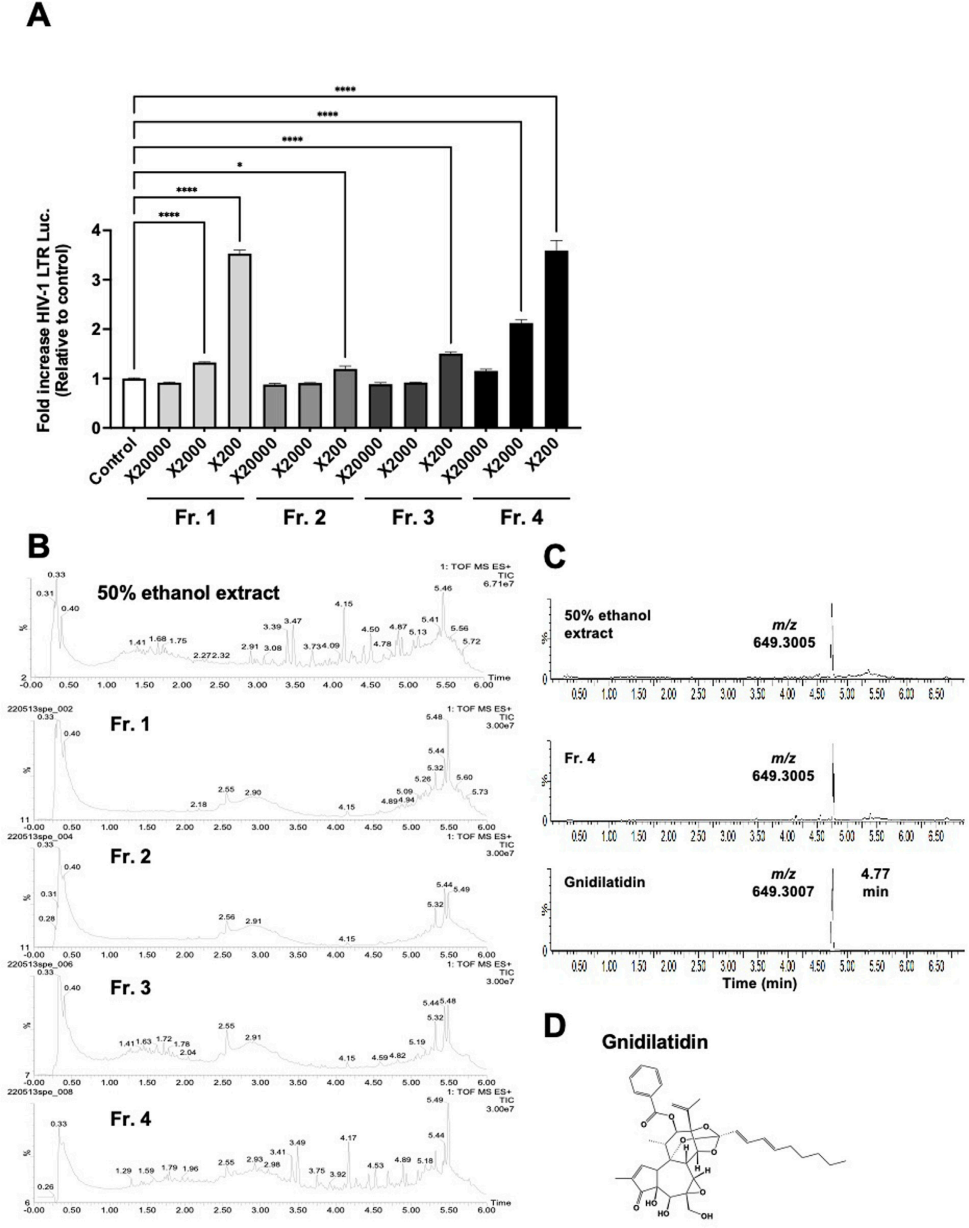
Estimation of active compounds in *Gnidia kraussiana*, G.K. **(A)** Transcriptional activity from HIV-1 LTR induced by fractionated samples. Samples of each fraction were evaluated using TZM-bl cells by dilution as indicated. Control corresponds to 0.5% DMSO. Statistical analysis was performed using one-way ANOVA with Dunnett’s multiple comparisons test in GraphPad Prism. *P < 0.05, ****P < 0.0001. Each value represents the mean ± S.D of three experiments. **(B)** The Total Ion Chromatograms (TICs) of the 50% ethanolic extract and fractions 1–4 shows the overall chemical profile of the extract. **(C)** Extracted Ion Chromatograms (EICs) obtained from UHPLC-HRMS analysis of (i) 50% ethanolic extract of *G. kraussiana*, (ii) active fraction 4, and (iii) commercially available gnidilatidin standard. The peak of interest showed an experimental m/z value of 649.3005, closely matcTIChing the theoretical value of 649.3007, confirming the identity as gnidilatidin. **(D)** Chemical structure of proposed compound identified using dictionary of natural products online database.

To isolate the active compound in *G. kraussiana*, the 50% ethanolic extract of *G. kraussiana* was fractionated as shown in Materials and Methods, and each fraction was evaluated for latency-reversing activity using TZM-bl cells. In this fractionation system, Fraction 4 was the least polar fraction. Fractions 1 and 4 showed significant latency-reversing activity (Figure 2A), and were analyzed in detail using UHPLC-HRMS. To provide an overview of the extract and fractions of *G. kraussiana*, Total Ion Chromatograms (TICs) were generated for both the 50% ethanolic extract and each fraction (Figure 2B). Although multiple peaks were detected in fraction 1, we were unable to conclusively identify specific compounds based on the available spectral data. In contrast, a peak specific to both the 50% ethanolic extract and Fraction 4 was clearly observed. This peak appeared at m/z 649.3005 ([M + H]^+^), which closely matched the theoretical value of 649.3007, confirming the molecular formula C_37_H_44_O_10_. A search using the Dictionary of Natural Products (CRC Press, online version) suggested that this compound is gnidilatidin (Figure 2C). To further confirm this identification, we compared the retention time of the peak in fraction 4 with that of a commercially available gnidilatidin standard using the extracted ion chromatogram (EIC). As shown in Figure 2C, the peak in Fraction 4 and the standard exhibited identical retention times. Based on these results, and supported by both TIC and EIC data, we concluded that gnidilatidin is one of the active compounds in *G. kraussiana* responsible for the observed latency-reversing activity (Figure 2D).

### 3.3 Gnidilatidin shows activity as LRA

**FIGURE 3:**
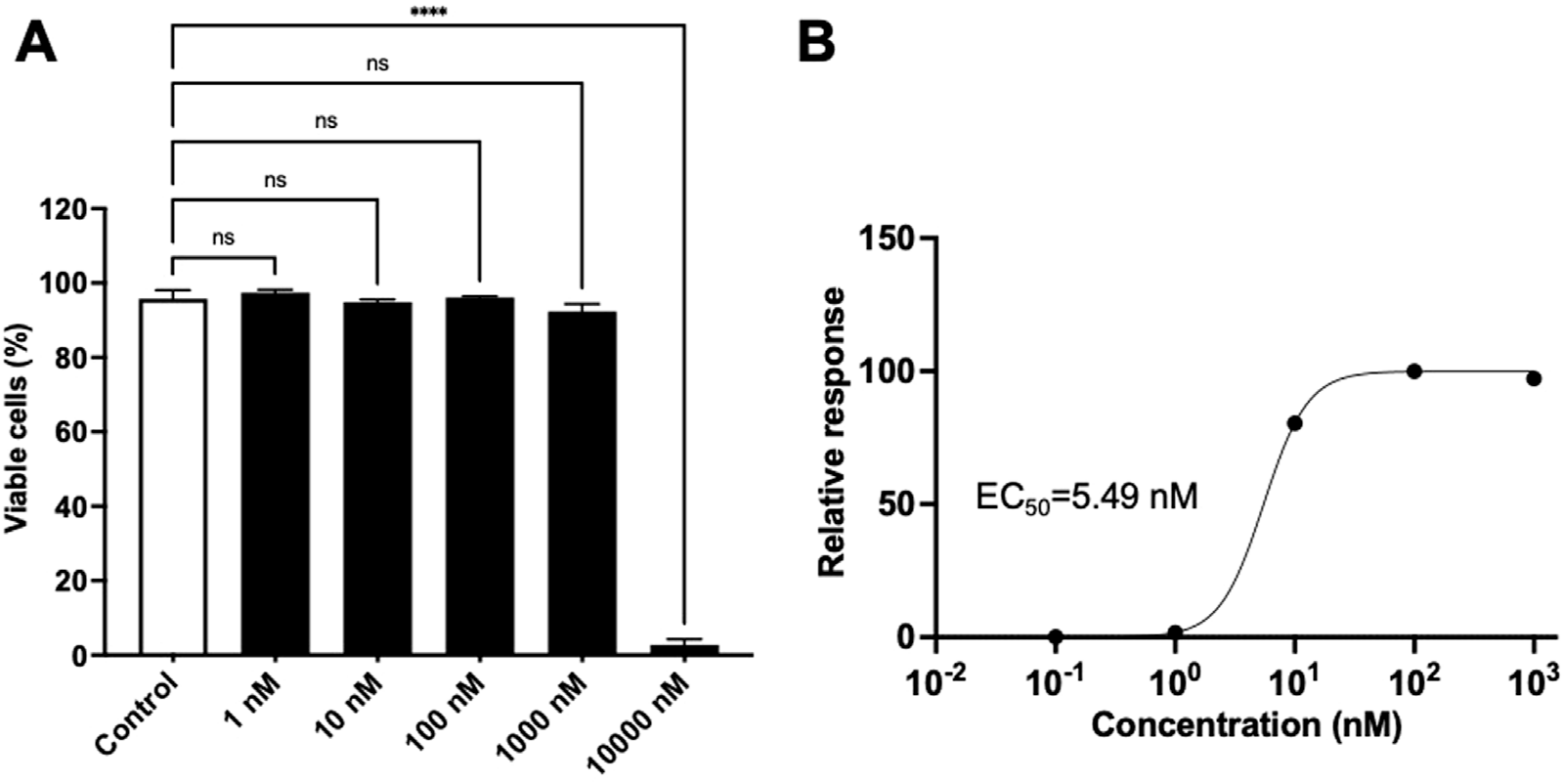
Effects of gnidilatidin in J-Lat 10.6 cells.**(A)** Cytotoxicity of gnidilatidin. J-Lat 10.6 cells were treated with various concentrations of gnidilatidin for 24 h, and cytotoxicity was assessed by flow cytometry. **(B)** Dose-dependent latency-reversing activity of gnidilatidin. J-Lat 10.6 cells were treated with gnidilatidin or prostratin for 24 h, and the percentage of GFP-positive cells was measured by flow cytometry. Control corresponds to 0.5% DMSO. Statistical analysis was performed using one-way ANOVA with Dunnett’s multiple comparisons test (GraphPad Prism). ****P < 0.0001; ns, not significant. Each value represents the mean ± S.D of three experiments.

The LRA activity of commercially obtained gnidilatidin was evaluated using J-Lat 10.6 cells. As shown in Figure 3A, gnidilatidin showed no toxicity at concentrations as high as 1,000 nM. On the other hand, gnidilatidin exhibited an EC_50_ of 5.49 nM in J-Lat 10.6 cells (Figure 3B). We previously reported an EC_50_ of 720 nM for prostratin using the same evaluation system (Yanagihara et al., 2021), indicating that gnidilatidin is more effective than prostratin. These findings suggest that gnidilatidin has the potential to act as an LRA and may contribute to eradicating the virus from its latent host reservoir cells.

### 3.4 Gnidilatidin downregulates CXCR4

**FIGURE 4:**
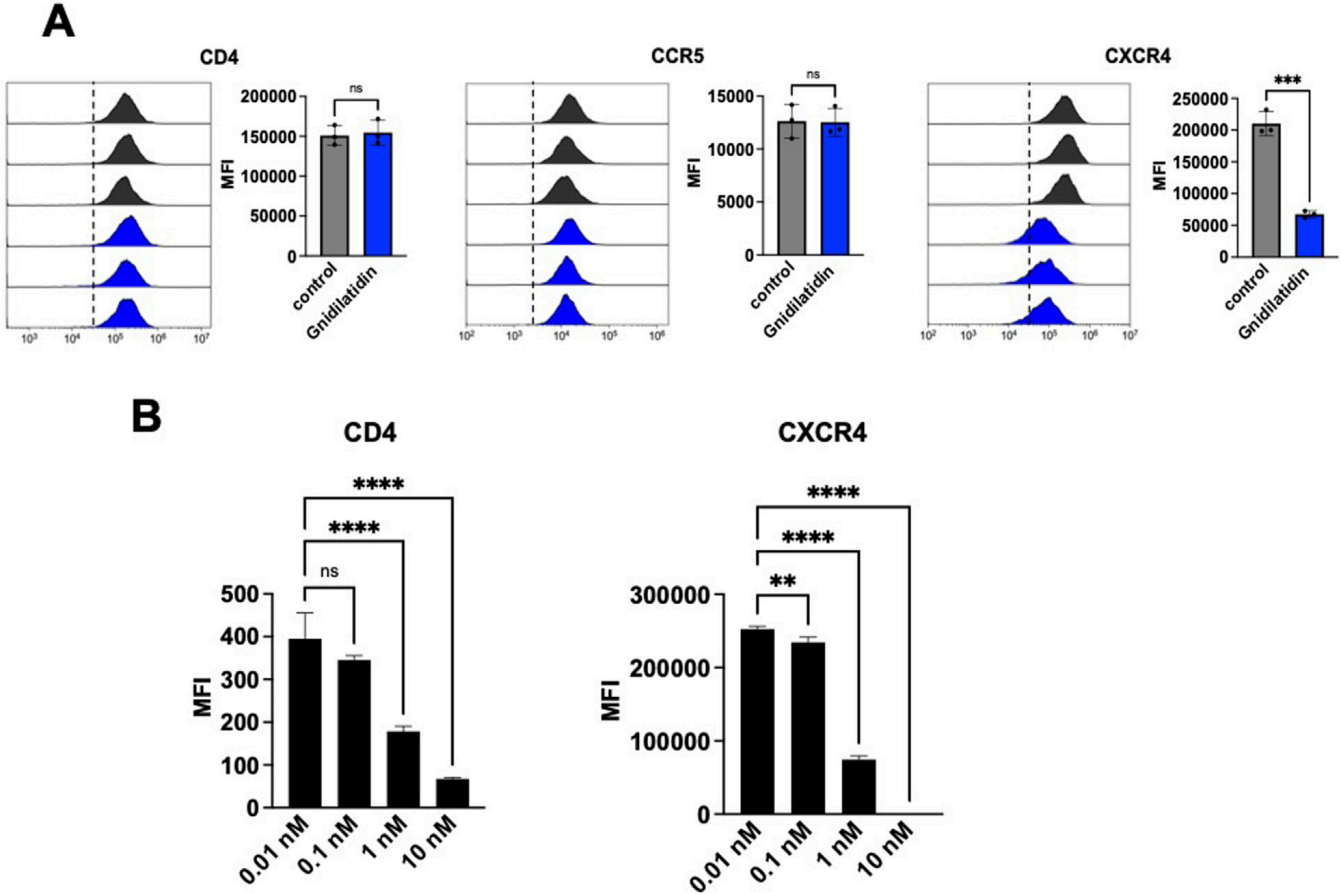
Analysis of expression levels of HIV-1 entry receptors. The surface expression levels of HIV-1 entry receptors of **(A)** TZM-bl cells and **(B)** Jurkat cells were analyzed by flow cytometry. Cells were treated with gnidilatidin for 48 h. Control corresponds to 0.5% DMSO. Statistical analysis was performed by unpaired t-test (GraphPad Prism) **P < 0.01; ***P < 0.001; ****P < 0.0001; ns, not significant. Each value represents the mean ± S.D of three experiments.

Finally, we evaluated whether gnidilatidin not only activates HIV-1 latent infection but also inhibits HIV-1 replication. Huang et al. have reported that gnidimacrin, a PKC activator isolated from the roots of *Stellera chamaejasme* L., can activate HIV latency and also downregulate the HIV-1 entry receptor (Huang et al., 2011). Thus, we have also evaluated the effect of gnidilatidin on the expression of CD4, CCR5, and CXCR4 in TZM-bl cells. As shown in Figure 4A, the fluorescence intensity in the flow cytometer analysis revealed that the treatment with 10 nM gnidilatidin efficiently downregulated the expression of the CXCR4 chemokine receptor, but did not affect CD4 or CCR5. In addition, gnidilatidin downregulated the expression of CD4 and CXCR4 in Jurkat cells in a concentration-dependent manner (Figure 4B). These findings suggest that in situations where CXCR4-directed HIV-1 is predominant in the body, gnidilatidin may be of considerable value in facilitating a “shock and kill.”

## 4 Discussion

The therapeutic potential of indigenous medicinal plants has long been recognized as a cornerstone of traditional healthcare in many regions, globally. In Sudan, traditional healthcare practices have been rooted on centuries of experiential knowledge. They provide essential treatment options for many rural communities where access to modern medical resources is limited. In light of these traditions, our study represents an important first step in the systematic evaluation of the efficacy of traditional Sudanese medicinal plant preparations, particularly in the context of HIV/AIDS therapy.

In this study, we initially evaluated ten Sudanese medicinal plants for their potential to activate HIV-1 transcription. Among them, both the absolute methanolic and 50% ethanolic extracts from *A*. *senegalensis, L*. *arborea, F*. *cretica, G*. *chrysantha, and G*. *kraussiana* demonstrated notable transcriptional activation from the HIV-1 long terminal repeat (LTR). Interestingly, the 50% ethanolic extract of *G*. *kraussiana* exhibited the most pronounced dose-dependent activity, with an EC_50_ value of 3.34 μg/mL. This high potency suggested that *G. kraussiana* is a promising source of LRA and prompted further bioactivity-based fractionation methods to elucidate its active components.

Subsequent UHPLC-HRMS analysis led to the identification of gnidilatidin, a daphnane diterpenoid, in both the crude *G. kraussiana* extract and in Fraction 4. Gnidilatidin is a PKC activator structurally similar to other LRAs such as gnidimacrin, prostratin, and ingenol-3-angelate. These findings strongly suggest that gnidilatidin is a key bioactive constituent driving the observed HIV-1 LTR transcriptional activation; however, the possible contribution of other compounds or synergistic interactions remains to be clarified through further studies using modified extraction approaches. Interestingly, in contrast to gnidilatidin, compounds such as gnidimacrin, prostratin, and ingenol-3-angelate, downregulate CD4, CCR5, and CXCR4 (Kulkosky et al., 2001; Huang et al., 2011; Jiang et al., 2015). When reversal of latency by gnidilatidin and prostratin was investigated in J-Lat 10.6 cells, gnidilatidin (EC_50_ = 5.49 nM) had a higher LRA activity than prostratin (EC_50_ = 720 nM). In addition, the effect of gnidilatidin appeared to be cell-type-dependent, with the downregulation of CXCR4 expression observed in TZM-bl cells (a HeLa cell clone), but the downregulation of CD4 and CXCR4 expression observed in Jurkat-derived J-Lat10.6 cells. This dual functionality makes gnidilatidin a promising candidate for advancing the “shock and kill” strategy, which aims to reactivate latent HIV-1 for immune clearance while suppressing viral replication. Understanding the mechanisms underlying gnidilatidin’s action will be critical to optimizing its therapeutic potential in combination with existing cART regimens.

Notably, a structurally identical compound to gnidilatidin, yuanhuacin, was recently isolated from the dichloromethane extract of *Gnidia sericocephala* roots collected in Tugela Ferry, Msinga region, KwaZulu-Natal, South Africa (Tembeni et al., 2022). Their study reported both anti-HIV activity and LRA potential of yuanhuacin, which was identified through a research flow similar to ours: the plant was selected based on its use by traditional health practitioners for HIV/AIDS management, as noted in their statement, “This plant species was selected based on its use by traditional health practitioners for HIV/AIDS management.” This parallel independently validates the ethnopharmacological approach we employed in Sudan. However, to our knowledge, their study did not report effects of yuanhuacin on host cell receptors such as CD4 or CXCR4. In contrast, our study is the first to demonstrate that gnidilatidin downregulates both CD4 and CXCR4 expression, a novel finding that adds mechanistic insight to the potential utility of this compound in HIV-1 “shock and kill” strategies.

To further explore why gnidilatidin exhibits the ability to downregulate CD4 and CXCR4, we considered its structural features that may underlie these biological effects. The presence of an epoxide moiety in gnidilatidin, as similarly seen in gnidimacrin and related daphnane diterpenes, may play a critical role in its biological activity (Borris and Cordell, 1984). In particular, the 6α, 7α-epoxy is a characteristic structure in 6-epoxy and 1-alkyldaphnane diterpenoids, and it has been reported to enhance anticancer activity. For example, gnidilatidin (yuanhuacin), yuanhuadin, yuanhuafin, and yuanhuapin generally showed stronger cytotoxicity against A549 cells compared to genkwanine A-L derivatives possessing a 6α, 7α-dihydroxyl group (Zhan et al., 2005). Additionally, genkwadane B exhibited stronger activity across seven cancer cell lines than genkwadane C, suggesting that the opening of the 6α, 7α-epoxy ring may negatively impact antineoplastic activity (Li et al., 2013). These structural features may facilitate the selective activation of PKC isoforms, which in turn may modulate membrane receptor expression, including that of CD4 and CXCR4, as observed in this study. Epoxides, while contributing to conformational rigidity and potential binding interactions with cellular PKC isozymes, are also chemically reactive groups that can form covalent adducts with nucleophilic residues in proteins, potentially contributing to both biological activity and cytotoxicity. Thus, careful consideration of this moiety may be necessary in further derivatization and drug development efforts.

In conclusion, this study substantiates the ethnopharmacological relevance of *G*. *kraussiana* and provides scientific validation for its traditional use in managing HIV/AIDS-related conditions. The identification of gnidilatidin as a bioactive constituent capable of inducing HIV-1 transcription highlights its potential as a lead compound in latency-reversing strategies. The present findings establish a robust foundation for further exploration of the mechanistic basis, safety, and pharmacokinetic properties of gnidilatidin. Subsequent research should focus on optimizing its activity profile and assessing its efficacy in combination with cART, thereby contributing to the principal objective of achieving a practical cure for HIV-1.

## Data Availability

The original contributions presented in the study are included in the article/supplementary material, further inquiries can be directed to the corresponding author.

## Abbreviations

HIV-1, human immunodeficiency virus type 1; cART, combination antiretroviral therapy; LRA, latency-reversing agent; LTR, long terminal repeat; LMICs; low- and middle-income countries.
